# Correction: A novel mutation in the *SLCO2A1* gene, encoding a prostaglandin transporter, induces chronic enteropathy

**DOI:** 10.1371/journal.pone.0247691

**Published:** 2021-02-19

**Authors:** Keisuke Jimbo, Toshiaki Okuno, Ryuichi Ohgaki, Kou Nishikubo, Yuri Kitamura, Yumiko Sakurai, Lili Quan, Hiromichi Shoji, Yoshikatsu Kanai, Toshiaki Shimizu, Takehiko Yokomizo

The metabolic pathways of PGE_2_ and PGF_2_α in the original Fig 2A have been combined in the updated version of Fig 2A.

Fig 3C is missing an explanation of the representation of the black circle. The authors have provided the corrected version here.

**Fig 2:**
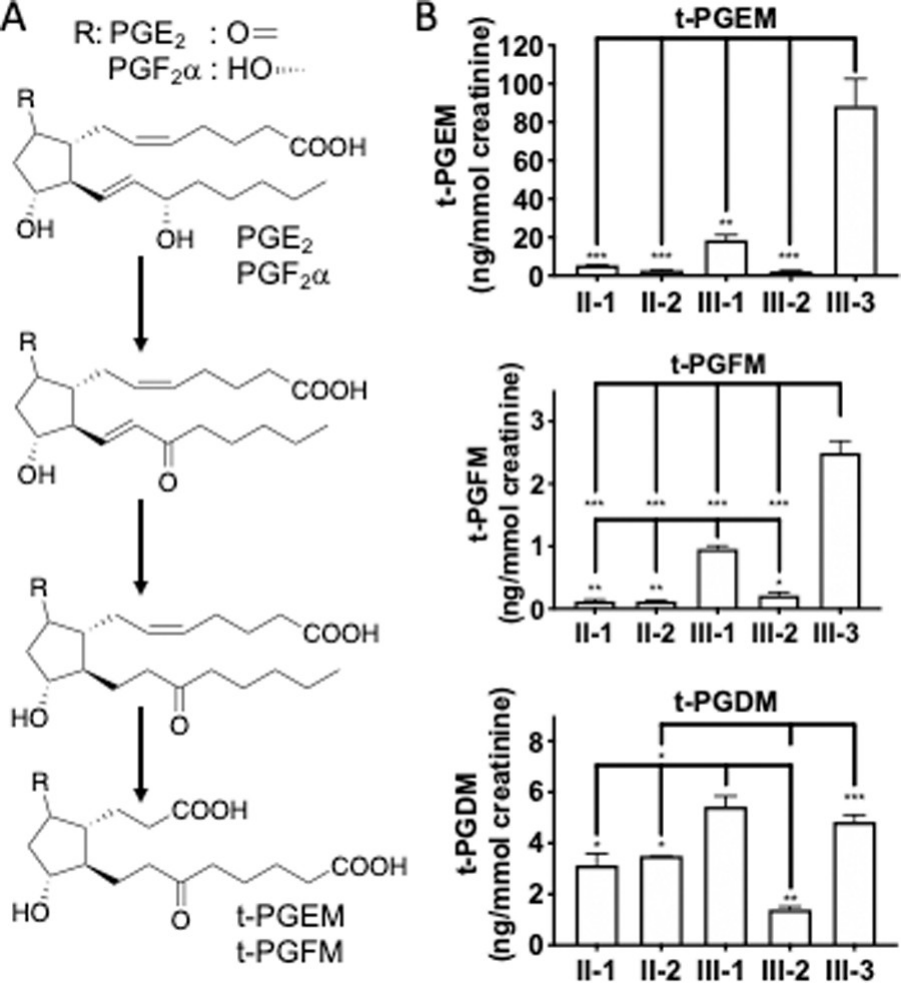
Urinary levels of tetranor-prostaglandin metabolites, t-PGEM, t-PGFM, and t-PGDM. (**A**) Metabolic pathways of prostaglandin E_2_ and prostaglandin F_2_α. (**B**) Urinary t-PGEM, t-PGFM, and t-PGDM were measured using UPLC-MS/MS. Each bar represents the mean ± SEM (n = 3). ***, *p* < 0.0001; **, *p* < 0.001; *, *p* < 0.01 (One-way ANOVA and Tukey’s multiple comparisons test).

**Fig 3:**
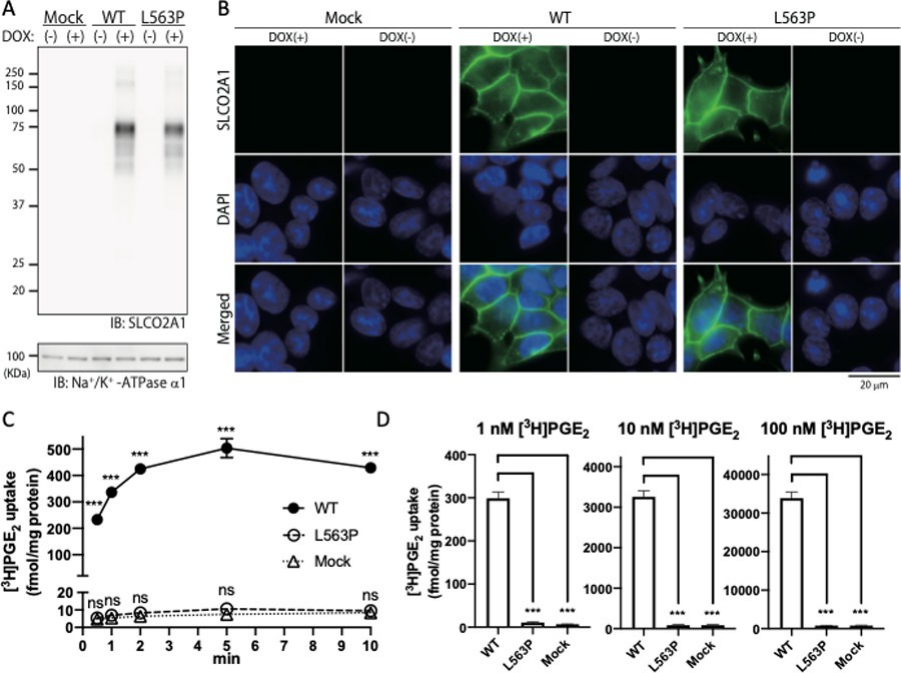
Expression, subcellular localization, and PGE_2_ uptake activities of SLCO2A1 and the L563P mutant. (**A**) Membrane fractions of Flp-In T-REx 293 cells expressing WT-SLCO2A1 and the L563P mutant were subjected to western blotting analysis. (**B**) Cells were treated with doxycycline (DOX) for 24 hr and fixed. Immunofluorescence staining was performed with anti-SLCO2A1 antibody and Alexa Fluor 488-conjugated goat anti-rabbit IgG. (**C**) Cells were treated with DOX, and then incubated for the indicated periods with 1 nM [^3^H]PGE_2_. After extensive washing with HBSS, the cells were lysed with 0.1 N NaOH and the radioactivity was measured. Data represent the mean ± SEM (n = 4). ***, *p* < 0.0001, ‘ns’, not significant. (One-way ANOVA and Tukey’s multiple comparisons test). (**D**) Cells were treated with DOX and incubated for 1 min with 1, 10, and 100 nM [^3^H]PGE_2_. The radioactivity was measured as described above. The uptake values were normalized by the protein concentrations in the cell lysates. Experiments were repeated three times, with quadruplicates for each sample (n = 4). Each bar represents the mean ± SEM. ***, *p* < 0.0001 (One-way ANOVA and Tukey’s multiple comparisons test).
