# Supplementary material for: A novel mutation in the SLCO2A1 gene, encoding a prostaglandin transporter, induces chronic enteropathy
Source: PLoS One. 2020 Nov 9;15(11):e0241869. doi: 10.1371/journal.pone.0241869 (PMC7652309; doi:10.1371/journal.pone.0241869)
Supplement: S1 Table — (DOCX) [file pone.0241869.s001.docx]

**S1 Table.** **Primers for mutation analysis of the SLCO2A1 gene**

| Amplicon | Forward (5′–3′) | Reverse (5′–3′) | Size (bp) |
| --- | --- | --- | --- |
| Exon 1 | GCGTTTCATCATCGGCGG | CTCCGGCAGACAGAAGCG | 360 |
| Exon 2 | CGAGGAAACAGAGGTCAGCC | AGCTCAGTACCTGGCACAAG | 450 |
| Exon 3 | GTCTTCTCTGTGGGTGGGTG | AATCACAGCAGGAGGTGAGC | 438 |
| Exon 4 | CAAGGGGCCTGGGAGAAAG | CAGCTGGGAGGTAATGGAGC | 496 |
| Exon 5 | GGAAGTTGGCTGGAGGAAGA | GGGATGAGTGAGCGAGTGAG | 450 |
| Exon 6 | GCTGTGCTTCATGTGCAGTG | TCTACCCCCACATCCCTTTC | 409 |
| Exon 7-8 | AGACTCAAGGGCTCAGGGAA | CTCTGGGCTCACCAATGAGG | 522 |
| Exon 9 | GGCAAGGCAATCCTGTCTCA | AAGGAAGCAGGAAGGAAGATGT | 487 |
| Exon 10 | TTGGCTCCACCAGGCATAAT | GGTTGGATGTGGCTCAGAGG | 484 |
| Exon 11 | GCCCAAACAGTGACAGAGGT | ACTCTGAGGGCCACAAAAGG | 486 |
| Exon 12 | CATCAGGGCTGGTTCTGACC | GGCAGGAATGCAAAGTTTTGTTC | 505 |
| Exon 13 | CCCAGGTCCCTTTTGTCTTCT | AGAGGTGGCCCTTCATGTTC | 466 |
| Exon 14 | GAGTCCCATGCCTACTCCCT | GGAACTGTGGGGAGGACTCT | 499 |
