# Supplementary material for: A novel mutation in the SLCO2A1 gene, encoding a prostaglandin transporter, induces chronic enteropathy
Source: PLoS One. 2020 Nov 9;15(11):e0241869. doi: 10.1371/journal.pone.0241869 (PMC7652309; doi:10.1371/journal.pone.0241869)
Supplement: Attachment — Submitted filename: Response_to_ Reviewers.docx [file pone.0241869.s002.docx]

**Point-by-point Responses**

***Reviewer #1:*** *Jimbo et al. reported a sibling case with chronic enteropathy associated with SLCO2A1 gene (CEAS) and found a new missense variant (c.1688T>C) in the SLCO2A1 gene. They also confirmed that this mutation causes loss of transporter function by the PGE2 uptake experiment.*

*Major point
1. Clinical features about the patients are unclear. For example, there is no description of patient III-1 and the location of the small intestinal lesions of patient III-3 is unclear. They should describe in detail the gastrointestinal and extraintestinal features of the patients. If possible, they should discuss the difference between siblings.*

Response: Thank you very much for your critical comments. We described clinical features about the patients as follows:

P6L86: However, we observed multiple circular and tape-like shallow ulcers in the entire small intestine and predominantly in the lower ileum using intestinal capsule endoscopy, strongly indicating CEAS (Fig. 1B).

P7L98: Through genetic analysis of the parents and siblings, we identified the same heterozygous variants in the *SLCO2A1* gene of patient III-1, who had past medical history of recurrent abdominal pain, but it had not been scrutinized at medical institutes. After the identification of the genetic mutation, she was found to have hypoproteinemia and anemia similar to patient III-3 by laboratory test. She also exhibited multiple shallow and tape-like ulcers in entire small intestine and predominantly in the lower ileum by capsule endoscopy (Fig. 1B).

*Minor point
1. P6L74. The authors wrote, "the disease can now be genetically diagnosed...". but CEAS is diagnosed by clinical features and a genetic test and genetic test just help in diagnosing. Please revise this sentence.*

Response: Thank you for your critical comment. We revised our manuscript as follow.

P6L74: the disease is diagnosed by clinical features and a genetic test in the *SLCO2A1* gene.

*2. P6L81. The word "consanguineous" should be wrong. Please make sure.*

Response: Thank you for your useful comment. We modified our manuscript as follow.

P6L81: to healthy non-consanguineous parents (Fig. 1A).

*3. P9L120. The word "CNSU" should be changed to CEAS.*

Response: Thank you for your useful comment. We modified our manuscript as follow.

P9L127: in the CEAS patients were significantly higher

*4. P10L150. There is no explanation about the representation of the black circle and so on in the Fig 3c.*

Response: Thank you for your useful comment. We added the representations of the black circle, open circle, and open triangle in the Fig 3c.

We thank Reviewer #1 for the constructive and insightful comments, which helped us to substantially improve our manuscript.

***Reviewer #2:*** *This is a new case report for the sibling of CEAS with compound heterozygous variants (c.940+1G>A and c.1688T>C). Jibo et al found a novel SLCO2A1 mutation (c.1688T>C) and confirmed that the SLCO2A1 variant (L563P) had no PGE2 transport activity when it was expressed in Flp-In T-Rex 293 cells. The structure model of SLCO2A1 suggests the substitution of Leu with Pro impairs the transport function. This paper is well-described, and molecular mechanism supports their clinical observation. Information on clinically associated mutations of SLCO2A1 is worthwhile to be shared for efficient genetical diagnosis. The following minor concerns are raised.*

*1. Figure 2A shows the metabolic pathway of both PGE2 and PGF2a; however, they are basically the same. They should combine them and show only essential parts of the pathway.*

Response: Thank you for useful comments. We have combined the metabolic pathway of PGE2 and PGF2a in Figure 2A.

*2. SLCO2A1 defects also cause PHO/PDP. Have the authors observed any clinical features of PHO/PDP in these patients? It would be appreciated to describe or mention clinical manifestations of PHO/PDP.*

Response: Thank you for critical comments. We consulted two CEAS patients to a dermatologist in our hospital and got the answer that there were no characteristic findings of PHO/PDP. Therefore, we modified our manuscript as follows.

P7L104: (Fig. 1B). In addition, because *SLCO2A1* defects also cause another disease primary hypertrophic osteoarthropathy (PHO)/pachydermoperiostosis (PDP), we consulted two CEAS patients to a dermatologist in our hospital and got the answer that there were no characteristic findings of PHO/PDP.

*3. In Figure 4, the authors suggest that L563 side chain is not directly involved in substrate biding because the side chain is outward (line 168). Does this mean that the side of the leucine outward from the helix does not affect substrate binding? This description is a little bit hard to understand. It would be nicer If the authors clarified this molecular basis more clearly.*

Response: Thank you for useful comments. We consider that the side chain of the leucine outward from the helix is not involved in substrate binding. We modified the manuscript as follows:

P12L172: The substrate may occupy a pocket between the transmembrane helixes similar to that of GlpT.

We thank Reviewer #2 for the constructive and insightful comments, which have helped us to substantially improve our manuscript.
